# Psychological Resilience and Adverse Mental Health Issues in the Thai Population during the Coronavirus Disease 2019 Pandemic

**DOI:** 10.3390/ijerph192013023

**Published:** 2022-10-11

**Authors:** Chidchanok Ruengorn, Ratanaporn Awiphan, Chabaphai Phosuya, Yongyuth Ruanta, Nahathai Wongpakaran, Tinakon Wongpakaran, Kednapa Thavorn, Surapon Nochaiwong

**Affiliations:** 1 Department of Pharmaceutical Care, Faculty of Pharmacy, Chiang Mai University, Chiang Mai 50200, Thailand; 2 Pharmacoepidemiology and Statistics Research Center (PESRC), Chiang Mai University, Chiang Mai 50200, Thailand; 3 Department of Psychiatry, Faculty of Medicine, Chiang Mai University, Chiang Mai 50200, Thailand; 4 Ottawa Hospital Research Institute, Ottawa Hospital, Ottawa, ON K1H 8L6, Canada; 5 ICES uOttawa, Ottawa, ON K1Y 4E9, Canada; 6 School of Epidemiology and Public Health, Faculty of Medicine, University of Ottawa, Ottawa, ON K1G 5Z3, Canada

**Keywords:** anxiety, coping, COVID-19, depression, mental health, psychological resilience, stress, well-being

## Abstract

In light of the coronavirus disease 2019 (COVID-19) pandemic and the enormous amount of uncertainty caused by it, mental health issues have become a great concern. Evidence regarding the effects of psychological resilience on the Thai population is scarce. We evaluated psychological resilience during the first wave of the COVID-19 pandemic and its association with the risk of mental health outcomes, such as depression, anxiety, stress, and health-related well-being. This cross-sectional study was a part of the HOME-COVID-19 project, which conducted an online survey of 4004 members of the general population in Thailand using the Brief Resilience Coping Scale. Logistic regression was performed to identify the association between psychological resilience and mental health issues and well-being. Groups with prevalence rates of 43.9%, 39.2%, and 16.9% were classified as low, moderate, and high resilient copers, respectively. Using high resilient copers as a reference group, the low resilient copers had a higher chance of having mental health adversities. The adjusted odds ratio (OR) was 1.89 (95% confidence interval [CI], 1.39–2.56; *p* < 0.001) for depression, 2.13 (95% CI, 1.45–3.14; *p* < 0.001) for anxiety, 4.61 (95% CI, 3.30–6.45; *p* < 0.001) for perceived stress, and 3.18 (95% CI, 2.31–4.38; *p* < 0.001) for low well-being. For the medium resilient copers, only low well-being was found to be statistically significant (OR, 1.60; 95% CI, 1.16–2.20; *p* = 0.004). It is important that resilience be considered in the development of strategies for managing the COVID-19 pandemic to prevent or reduce adverse mental health outcomes.

## 1. Introduction

The coronavirus 2 (SARS-CoV-2) disease termed as coronavirus disease 2019 (COVID-19) pandemic has impacted the well-being of global populations in different ways across the world, and the degrees of vulnerability have varied among different communities [1]. Evidence has shown some of the major COVID-19-related stressors to be disease infection, loss of loved ones, physical distancing, secondary adversities, such as economic crises, and psychosocial effects [2,3]. The psychological consequences of the pandemic have become a great concern in terms of its short- and long-term impacts on mental health. As of June 2020, our systematic review and meta-analysis results regarding the contemporary global prevalence of mental health issues among the general population amid the COVID-19 pandemic estimated a global prevalence of 28.0% for depression, 26.9% for anxiety, 24.1% for post-traumatic stress symptoms, 36.5% for stress, 50.0% for psychological distress, and 27.6% for sleep problems, and variations in disparities have been widely found across countries and regions [1]. Before the COVID-19 pandemic, it is estimated that 2.9% and 1.7% of female and male Thai people suffered from depression [4]. However, during Thailand’s first wave of the COVID-19 pandemic, this number had increased to 25.6%, 9.7%, and 6.0% for mild, moderate, and severe depressive symptoms, respectively [2].

Resilience has attracted research attention for decades; however, studies vary substantially in their definitions and measurements. Resilience is defined as a process comprising immunity and stability undisturbed by a prolonged period of adversity, bouncing back from adversity, the ability to regain mental strength after a stressful period or event, and growth, meaning that the person performs even better after experiencing adversity [5]. Several researchers have defined resilience as a trait, process, and outcome [6]. Theoretically, resilience can be categorized into physical, emotional, economic, and psycho-social aspects [7]. Psychological resilience coping, which has attracted the attention of researchers over the past decade, has been suggested as a protective factor for prevention and management of the negative consequences of adversity when there are catastrophic events and substantial stressors [5,8,9]. It is a direct measure of the ability to adapt and cope, and in the present study, we tried to capture the tendencies to cope with stress in a highly adaptive manner using the Brief Resilience Coping Scale (BRCS) [10].

In the research on resilience and mental health issues, one previous systematic review conducted in all age groups of the population in 60 countries revealed the positive effect of state resilience on adaptation to stress. They also concluded that resilience is negatively associated with depression and anxiety. The authors also revealed positive correlations between resilience and life satisfaction and positive psychological effects [11]. In Thailand, during the COVID-19 pandemic, factors related to the psychological impact on older adults were investigated. The results showed that fear, anxiety, stress, loneliness, sense of control, emotional management, religion, and wisdom were among the psychological consequences of lower resilience. In addition, the quality of life was also affected in copers with lower resilience among older adults [12].

In light of the widening crisis and numerous uncertainties surrounding the pandemic, the burden of mental health and psychosocial problems has become a concern. People differ widely in their responses to challenges and difficulties. Psychological resilience is vital for the ability to cope effectively with hardship, uncertainty, and change [9]. Because resilience is an active process, the correlates of psychological resilience should be evaluated in the face of pandemics in Thailand, where evidence regarding the effects of psychological resilience is scarce. The present study evaluated the psychological resilience coping status during the first wave of the COVID-19 pandemic in the general Thai population and its association with the risk of mental health outcomes, including depression, anxiety, stress, and health-related well-being.

## 2. Materials and Methods

### 2.1. Study Design and Participants

Based on the Health Outcomes and Mental Health Care Evaluation Survey: Under the pandemic situation of COVID-19 (HOME-COVID-19), this study is part of the nonprobability public survey wave I from 21 April to 4 May 2020 (details of pre-specified parameters are published elsewhere) [13]. An open, online survey was circulated via the SurveyMonkey^®^ platform, which could limit one-time participation per unique internet protocol address. Relevant links or QR codes were distributed to the eligible population using a convenience and snowball sampling via social media networks, including public websites, Facebook, LINE, Twitter, and Instagram. For the current study, a voluntary nationwide public survey was conducted through a convenient selection of the target population in Thailand. This included the general population who: (i) were Thai citizens, permanent residents, or non-residents with work permits aged 18 years or more at the date of the survey; (ii) were employed full-time before the national lockdown owing to the COVID-19 outbreak; and (iii) could read and communicate in the Thai language and access the internet. We excluded participants who did not complete the online survey or spent less than 2 min or more than 60 min on the survey.

This study was performed in accordance with the Declaration of Helsinki, as well as the amendments or comparable ethical standards. The ethics approval was obtained from the institutional review boards of the Faculty of Public Health (ET010/2020) and Faculty of Pharmacy (23/2563), Chiang Mai University. Written consent was obtained from all participants before completing the questionnaire. The reporting of this study was in line with the Strengthening the Reporting of Observational Studies in Epidemiology Statement [14] and the Improving the Quality of Web Surveys: The Checklist for Reporting Results of Internet E-Surveys [15].

### 2.2. Psychological Resilience, Mental Health Outcomes, and Psychological Measurement Tools

The main independent variables were psychological resilience and mental health outcomes of interest, including symptoms of depression, anxiety, perceived stress, and well-being, during the COVID-19 pandemic in Thailand. The Thai versions of the validated measurement tools were used to evaluate mental health and psychosocial outcomes in this study (Table 1) [10,13,16,17,18,19,20,21,22,23].

### 2.3. Covariates

Relevant covariates were examined, including (i) sociodemographic characteristics (age, sex, marital status, educational level, religion, occupation, region of residence, living status, personal income (THB/month), reimbursement schemes, history of mental illness, history of chronic non-communicable diseases (NCDs)) and (ii) the economic burden and issues related to the COVID-19 pandemic (income loss, self-reported financial problems, media exposure, confirmed cases in the community, quarantine status, working from home status).

### 2.4. Statistical Analyses

Based on a pre-specified protocol and the results of previous studies reporting the prevalence of mental health problems (i.e., depression, anxiety, and stress) during the COVID-19 pandemic, 3.3–75.5% of the general population was selected [1,13]. In this circumstance, the sample was calculated using the compensation for a design effect of 2.0 and a response rate of 60%. To obtain a statistical power of 80% and a type I error probability of 0.05, at least 1310 participants were required for this study.

Descriptive statistics were used and illustrated as frequency (percentage), mean (standard deviation [SD]), or median with a range (min–max), as appropriate. Baseline characteristic differences between the psychological resilience groups were tested using Fisher’s exact test for categorical data and analysis of covariance or the Kruskal–Wallis test for continuous data. The prevalence of adverse mental health issues regarding the resilient coping status was estimated with corresponding 95% confidence intervals (CIs).

For the primary analysis, multivariable logistic regression models were used to investigate the association between psychological resilience and the risk of adverse mental health issues (high resilient copers, reference group) after controlling for the covariates of each outcome. Moreover, an ancillary analysis was also performed using multivariable linear regression models to confirm the linear relationship between psychological resilience and the risk of adverse mental health issues. The effect estimates were expressed as odds ratios (ORs) or β coefficients, along with 95% CIs. Multicollinearity was tested using a variance inflation factor (VIF) value of ≥4 as a cut-off point for further investigation, and a VIF value of ≥10 indicated substantial multicollinearity, which required correction. 

For each analysis, three models were analyzed based on the level of confounder adjustment: (i) model 1, adjusted for age and sexual identity; (ii) model 2, which included model 1 plus marital status, education level, religion, occupation, region of residence, living status, personal income, reimbursement scheme, history of mental illness, history of chronic non-communicable diseases, income loss, and financial problems; (iii) model 3, which included all the variables in model 2 plus information exposure, confirmed cases in the community, quarantine status, working from home, fear of COVID-19, perceived risk of infection, stigma toward infection, and perceived social support.

All the prevalence and effect estimates were weighted based on the national population and the rate of internet use obtained from the National Statistics Office of the Thai Ministry of Information and Communication Technology. All analyses were performed using Stata, version 14.0 (StataCorp, LLC, College Station, TX, USA). Two-tailed tests with *p*-values less than 0.05 were considered statistically significant.

## 3. Results

### 3.1. Participant Characteristics

Among the 4322 potential participants screened in the first wave of the HOME-COVID-19 survey, 318 were unable to complete the set of mental health and psychological measurement tools and were excluded (Supplementary Figure S1). Therefore, 4004 participants with a mean age of 29.1 (10.8) years were included in this study, of whom 2619 were female, 3164 (79.0%) lived with family, and 9.0% had a history of mental illness. Additionally, 1664 (41.65) and 2012 (50.2%) participants experienced income loss and self-reported financial problems during the COVID-19 pandemic, respectively. Most participants had a moderate-to-severe fear of COVID-19, medium-to-high perceived risk of COVID-19 infection, and moderate-to-high stigma toward COVID-19; however, most reported having moderate-to-high perceived social support. Regarding psychological resilience, the overall mean resilient coping (BRCS) score was 13.9 (3.1), with a median of 14 (range, 4–20). Specifically, 1756 participants (43.9%) were classified as low resilient copers, followed by medium resilient copers (1570 participants, 39.2%), and high resilient copers (678 participants, 16.9%) (Table 2).

### 3.2. Prevalence of Mental Health Issues According to Resilient Coping Status

Table 3 summarizes the prevalence of adverse mental health issues according to the degree of psychological resilience. Resilient coping status was associated with the unadjusted prevalence of mental health issues (all *p*-values for difference <0.001). Statistical differences were found in the prevalence rate of adverse mental health issues of low resilient copers: 56.2% (95% CI, 53.0–59.4) for depression, 35.2% (95% CI, 32.1–38.4) for anxiety, 90.1% (95% CI, 89.1–92.5) for perceived stress, and 64.0% (95% CI, 60.9–67.1) for low well-being. In contrast, the prevalence rates of adverse mental health issues were more similar among high resilient copers: 29.5% (95% CI, 24.8–34.7) for depression, 15.1% (95% CI, 11.5–19.5) for anxiety, 56.7% (95% CI, 51.2–62.0) for perceived stress, and 26.1% (95% CI, 21.5–31.3) for low well-being.

### 3.3. Psychological Resilience and the Risk of Adverse Mental Issues

Using high resilient copers as a reference group, the relationship between low resilient copers and the risk of depression, anxiety, perceived stress, and low well-being revealed statistically significant differences in all aspects after adjusting for various sets of confounders (models 1, 2, and 3). With respect to the full model adjustment (model 3), the adjusted ORs were 1.89 (95% CI, 1.39–2.56; *p* < 0.001) for depression, 2.13 (95% CI, 1.45–3.14; *p* < 0.001) for anxiety, 4.61 (95% CI, 3.30–6.45; *p* < 0.001) for perceived stress, and 3.18 (95% CI, 2.31–4.38; *p* < 0.001) for low well-being (Table 4). For the medium resilient coper group based on the full model adjustment, we only found a statistically significantly higher risk of low well-being (OR, 1.60; 95% CI, 1.16–2.20; *p* = 0.004) compared with participants who were identified as high resilient copers. Meanwhile, no relationships were observed for the other aspects of mental health issues (Table 4). 

Moreover, ancillary analysis confirmed the linear relationship between psychological resilience and the risk of adverse mental health issues, particularly among low resilient copers (Table 5). However, the relationship between medium resilient copers and the risk of perceived stress was found to be statistically significant after multivariable linear regression models were used (β coefficient, 1.05; 95% CI, 0.34 to 1.77; *p* = 0.004 for model 3 adjustment).

## 4. Discussion

### 4.1. Overview of the Findings

Our study evaluated psychological resilience and found that the overall mean score on the BRCS was medium, with a mean of 13.9 and a median of 14 (range, 4–20). Almost half of our participants (43.9%) had a low level of resilience, followed by medium (39.2%), and high (16.9%). With regard to the association between the psychosocial resilience level and mental health adversities, depression, anxiety, perceived stress, and health-related well-being, low resilient copers had statistically significantly higher levels of depression, anxiety, and perceived stress, and statistically significantly lower levels of well-being compared with high resilient copers in all models. The results were consistent with the ancillary analysis when the treated outcomes were continuous variables. Meanwhile, the effects were not as strong among the medium copers, and the results were in line with the lower resilience copers for perceived stress and well-being outcomes. Depression and anxiety were positively correlated in all models but only in some models that showed statistical significance compared with high resilient copers. This suggests that the higher the resilience score amid the crisis from the COVID-19 pandemic, the better the mental health consequences and health-related well-being as a result of the dose–response relationship pattern in all outcomes.

### 4.2. Comparisons with Previous Research

The mean BRCS score was 13.9 (SD 3.1; range 4–20) in our study, which is in line with a previous study that measured psychological resilience during the first weeks of the COVID-19 lockdown in the United States, using the 25-item Connor-Davidson Risk Scale (CD-RISC), which showed a mean score of 66.8 out of 100 [9]. When calibrated to a range of 0–5, the mean score was similar to that of our results (3.3 vs. 3.5). Concordance results have also been observed among healthcare professionals. For example, nurses in Turkey who were responsible for the treatment and care of patients with COVID-19 had an average calibrated Brief Psychological Strength Scale score of 3.2 [24]. Another study revealed that the mean (SD) 10-item CD-RISC score among front-line physicians was 3.6 [25]. This score was lower that the reported normative data in the general United States population, which was 4.0/5 [26]. Specifically, the prevalence of high resilient copers in our study was the lowest among the three groups at 16.9%, which is close to the 18.3% of the “good state resilience” group in the Barcelona Resilience Survey for Mental Health COVID-19 (BRIS-MHC) project [27]. This suggests that the level of psychological resilience may have been adversely affected by the emerging crisis.

The overall prevalence rates of mental health adversities among the participants observed were 44.1% for depression, 26.1% for anxiety, 75.3% for perceived stress, and 47.5% for low well-being. Mental health issues had a worsening trend with lower resilience levels. Compared with global prevalence from our meta-analysis study, which showed 28.0% for depression, 26.9% for anxiety, 36.5% for stress, our population had a higher prevalence of depression and stress [1]. However, the discrepancies may be due to many factors, such as the time of data collection, geographical areas, and healthcare systems. Previous reports in the general population have shown that individuals without a mental health history had lower psychological well-being and greater levels of anxiety and depression during the COVID-19 pandemic than they did in the period preceding the pandemic [8,28].

The COVID-19 pandemic has affected both the physical and mental health of people worldwide. Psychological resilience is a crucial factor in the ability to cope effectively with the hardship, uncertainty, and change. Several studies have reported consistent findings regarding the association between resilience and mental health issues, as well as quality of life and well-being. For instance, psychological resilience was negatively correlated with worse depression, anxiety, and somatization symptom scores while controlling for confounding factors during the epidemic peak of COVID-19 in China [29]. Lower scores on the CD-RISC were clearly associated with more severe depression, suicidal ideation, and anxiety. Resilience has also been associated with greater fear due to the effects of COVID-19 [9,30]. Another study demonstrated that individuals with lower levels of resilience had higher levels of traumatic stress responses and psychological morbidity during the COVID-19 pandemic [31]. This may be because lower resilience copers experienced greater difficulty coping with the emotional challenges of the pandemic. A systematic review showed an intercorrelation of resilience and mental distress, mental health, depression, anxiety, and quality of life in older adults [12].

Previous studies have demonstrated an important mediating role of resilience in the relationship between adverse events and mental health outcomes. Many previous studies have identified significant indirect pathways between resilience and mental health adversities. Resilience essentially mediates the association between traumatic experiences and post-traumatic adjustment [32]. Consequently, it was found that COVID-19-related stressful experiences had an effect on acute stress disorder through the resilience pathway among college students [33]. In addition, resilience was found to be a significant mediator of multiple adverse life events and subjective physical and mental health [32].

### 4.3. Strengths and Limitations

To our knowledge, this is the first study to report the prevalence of COVID-19-related psychological resilience in a Thai population nationwide. This study was predominantly conducted using a large sample size. However, it has some limitations. First, due to the nature of cross-sectional study data collection, causal relationships between psychological resilience and mental health outcomes cannot be inferred. Second, the data used were only in the early phase of the pandemic, which may not represent other periods of the pandemic in which the circumstances have changed. Despite the argument that resilience can change over time and be adjusted by context, the results are believed to be only partially changed due to the nature of resilience, which is collective over time and may be only somewhat affected when confronting difficulties. Therefore, the associations revealed in this study could be beneficial for future emerging infectious diseases. Third, since open online surveys were the only way to collect our data, the generalizability of our results may be limited to only those with access to the internet; the majority of our participants were members of the younger population. Fourth, despite an ancillary analysis that was in line with the main analysis, the uncertainty with respect to exposure to COVID-19-related information, resilient coping perception, and the risk of COVID-19-related public stigma should be examined in further studies. Fifth, moderate resilience did not have different effects on mental health outcomes. This may be due to the cut-off points of the resilience score, which were not validated in our population, or a lack of statistical power. Lastly, research on the long-term effects of resilience is needed to confirm the results and the impact of psychological resilience.

### 4.4. Implications for Public Health and Future Research

The findings of this study and the previous literature suggest that psychological resilience should be prioritized as a primary public health issue during the COVID-19 pandemic. What are the effective interventions for individual cultivation or enhancement? Several studies have suggested that good social support, daily activities, such as exposure to the outdoors and sun light, and getting more exercise are all associated with greater resilience. Spiritual health is another facet that cannot be ignored because of its independent relationship with higher resilience scores [9]. A study conducted in Germany also revealed helpful strategies, such as maintaining a healthy lifestyle and social contacts, accepting anxiety and negative emotions, enhancing self-efficacy, and accessing useful medical information [34]. In the research conducted in Thailand, resilience building programs were recommended in one study on older adults to focus on gratitude and positive emotions, enhance self-efficacy, support resources, personal competence, and tolerance of negative affect through community activities, including mindfulness meditation, journaling, healthy eating, and discovering life’s purpose [12]. Front-line healthcare providers are a high-risk group for mental health issues due to their regular exposure to trauma or distress, and resilience enhancement programs may benefit this group. A recent systematic review and meta-analysis suggested that interventions using mindfulness or cognitive behavioral therapy techniques, especially a combination of both methods, appeared to enhance the measures of resilience [35]. Further, interventions can be provided through individual remote counseling or therapy, including online self-help approaches, with the generation of appropriate media content.

The recommendations for future research are as follows. First, the modifiable factors associated with resilience should be another major focus of future research that offers solutions that truly match the problem. Therefore, we recommended an updated study on the current situation when most of the general population received the COVID-19 vaccine. Second, resilience is theoretically categorized into several aspects, and we only captured the psychological characteristics using the practical four-item Thai-BRCS, which focuses on mental health outcomes. However, other aspects should be assessed for their direction and association with mental health issues as well as health-related well-being. Third, different cut-off points of the Thai-BRCS version should be verified and validated to ensure the best performance of the tool. Fourth, research on the correlation between resilience and mental health issues among healthcare professionals is highly warranted. Fifth, a subgroup study highlighted psychological resilience among populations with mental health diseases. Sixth, the role of psychological resilience as a mediator of mental health outcomes during the COVID-19 pandemic should be examined to understand these complex pathways. A better understanding of the pathways through which COVID-19-related independent factors are linked to mental adversities may allow for future tailored psychological behavioral interventions.

## 5. Conclusions

The prevalence of low psychological resilience was high (43.9%), and it was associated with mental health adversities, including depression, anxiety, perceived stress, and low health-related well-being. Although the effects were not as strong in the medium resilient copers compared with the high resilient copers, a gradient increasing trend was found in all adverse mental outcomes. Psychological resilience should be prioritized for the development of effective coping strategies in the Thai population during the COVID-19 pandemic, particularly given that the pandemic’s uncertainties and impacts are still ongoing.

## Figures and Tables

**Table 1:** Measurement tools of the survey.

Instrument	Description
Brief Resilient Coping Scale—4 items (BRCS-4)	The BRCS-4 consists of four items to capture the tendencies to cope with stress in a highly adaptive manner. The BRCS revealed satisfactory reliability (Cronbach’s α = 0.80) [13]. The total score ranges from 4 to 20, with a higher score indicating greater resilience to cope. For scale interpretation, the BRCS scores were categorized as low (≤13 points), medium (14–16 points), or high (≥17 points) resilience copers [10].
Patient Health Questionnaire—9 items (PHQ-9)	The PHQ-9 is used for measuring depressive symptoms and comprises nine items. The total score ranges from 0 to 27, with higher scores reflecting greater depression severity. The cut-off score for the depressive symptom group was ≥9. The PHQ-9 Thai version showed good psychometric properties, with a Cronbach’s α of 0.79 [16].
Generalized Anxiety Disorder Scale—7 items (GAD-7)	The GAD-7 is used for measuring worry and anxiety symptoms and comprises seven items. The total score ranges from 0 to 21, with higher scores indicating more severe anxiety. A cut-off point of ≥8 was used to identify the general population with anxiety symptoms [17]. The psychometric properties of this tool were excellent, with a Cronbach’s α of 0.92 [18].
Perceived Stress Scale—10 items (PSS-10)	The PSS-10 was used to measure the perception of stress with 10 items. The scores range from 0 to 40; higher scores indicate a higher degree of stress. A cut-off point of ≥14 was considered to indicate perceived stress. The PSS-10 has good psychometric properties, with a Cronbach’s α of 0.85 [19].
World Health Organization Five Well-Being Index—5 items (WHO-5)	The WHO-5 with five questions was used to measure health-related personal well-being, with a higher score indicating a high well-being index. A cut-off point of <50 points was considered a low well-being index. WHO-5 showed good psychometric properties, with a Cronbach’s α of 0.87 [20].
Fear of COVID-19	A numerical rating scale of 0–10 points was used to measure the degree of fear of COVID-19. The degree of fear was classified as none/minimal (0–3 points), moderate (4–6 points), and severe (7–10 points) [13,21].
Perceived risk of COVID-19 infection	A numerical rating scale of 0–10 points was used to measure the degree of perceived risk of COVID-19 infection. The degree of perceived risk was classified as none/minimal (0–3 points), moderate (4–6 points), and severe (7–10 points) [13,21].
Stigma toward COVID-19 infection—10 items (COVID-PSS-10)	The COVID-PSS-10 consists of 10 items to measure the stigma toward COVID-19 in a public health survey. The BRCS revealed satisfactory reliability (Cronbach’s α = 0.85). The degree of public stigma toward COVID-19 infection was classified as none/minimal (≤18 points), moderate (19–25 points), or high (≥26 points) [22].
Perceived social support: Multidimensional Scale of Perceived Social Support—12 items (MSPSS-12)	The MSPSS-12 consists of 12 questions to measure individual perceptions of external social support. This scale had excellent internal consistency, with a Cronbach’s α of 0.92. For scale interpretation, the MSPSS-12 score was categorized as low (≤35 points), medium (36–60 points), or high (≥61 points) perceived support [23].

Abbreviations: COVID-19, coronavirus disease 2019.

**Table 2:** Participant characteristics and the resilient coping status in Thailand ^†^.

Participant Characteristics	Overall (n = 4004)	Resilient Coping Status			
		Low Resilient Coper: BRCS ≤ 13 Points (n = 1756, 43.9%)	Medium Resilient Coper: BRCS 14–16 Points (n = 1570, 39.2%)	High Resilient Coper: BRCS ≥ 17 Points (n = 678, 16.9%)	*p*-Value
Resilient coping—BRCS score, mean (SD); median (range)	13.9 (3.1); 14 (4–20)	11.1 (2.0); 12 (4–13)	15.1 (0.8); 15 (14–16)	18.2 (1.2); 18 (17–20)	<0.001
Age in years, mean (SD); median (range)	29.1 (10.8); 25 (18–79)	28.0 (9.5); 24 (18–72)	29.3 (11.4); 25 (18–79)	31.2 (12.4); 27 (18–73)	<0.001
≤30 years	2659 (66.4)	1196 (68.1)	1055 (67.2)	408 (60.2)	<0.001
31–50 years	1088 (27.2)	497 (28.3)	394 (25.1)	197 (29.0)	
≥51 years	257 (6.4)	63 (3.6)	121 (7.7)	73 (10.8)	
Sexual identity					
Male	1231 (30.7)	506 (28.8)	500 (31.8)	225 (33.2)	0.192
Female	2619 (65.4)	1181 (67.3)	1012 (64.5)	426 (62.8)	
Others	154 (3.9)	69 (3.9)	58 (3.7)	27 (4.0)	
Marital status					
Single	3208 (80.1)	1430 (81.4)	1275 (81.2)	503 (74.2)	0.001
Married/domestic partnership	693 (17.3)	289 (16.5)	257 (16.4)	147 (21.7)	
Divorced/widowed/separated	103 (2.6)	37 (2.1)	38 (2.4)	28 (4.1)	
Education level					
Illiterate/primary school/junior high school	127 (3.2)	86 (4.9)	25 (1.6)	16 (2.4)	<0.001
Senior high school/diploma/high vocational	1893 (47.3)	880 (50.1)	740 (47.1)	273 (40.3)	
Bachelor’s degree	1559 (38.9)	667 (38.0)	612 (39.0)	280 (41.3)	
Higher education	425 (10.6)	123 (7.0)	193 (12.3)	109 (16.0)	
Religion					
Irreligion	375 (9.4)	174 (9.9)	130 (8.3)	71 (10.5)	0.074
Buddhist	3454 (86.2)	1492 (85.0)	1378 (87.8)	584 (86.1)	
Christian/Muslim/Others	175 (4.4)	90 (5.1)	62 (3.9)	23 (3.4)	
Occupation					
Unemployed/retired	391 (9.8)	200 (11.4)	128 (8.1)	63 (9.3)	<0.001
Employed	2024 (50.5)	851 (48.5)	791 (50.4)	382 (56.3)	
College student	1589 (39.7)	705 (40.1)	651 (41.5)	233 (34.4)	
Region of residence					
Capital city and its environs	1425 (35.6)	597 (34.0)	574 (36.6)	254 (37.5)	0.162
Non-capital city and its environs	2579 (64.4)	1159 (66.0)	996 (63.4)	424 (62.5)	
Living status					
Alone	576 (14.4)	246 (10.0)	231 (14.7)	99 (14.6)	0.978
With family	3164 (79.0)	1394 (79.4)	1234 (78.6)	536 (79.1)	
With others	264 (6.6)	116 (6.6)	105 (6.7)	43 (6.3)	
Person income, THB/month ^‡^					
≤10,000 (≤308 USD)	1905 (47.6)	908 (51.7)	719 (45.8)	278 (41.0)	<0.001
10,001–20,000 (309–616 USD)	1054 (26.3)	478 (27.2)	397 (25.3)	179 (26.4)	
>20,000 (>616 USD)	1045 (26.1)	370 (21.1)	454 (28.9)	221 (32.6)	
Reimbursement scheme					
Government/state enterprises	539 (13.5)	179 (10.2)	247 (15.7)	113 (16.7)	<0.001
Universal coverage scheme	1329 (33.2)	626 (35.6)	496 (31.6)	207 (30.5)	
Social security scheme	1161 (29.0)	534 (30.4)	443 (28.2)	184 (27.1)	
Self-payment/others	975 (24.3)	417 (23.8)	384 (24.5)	174 (25.7)	
History of mental illness					
No	3645 (91.0)	1558 (88.7)	1462 (93.1)	625 (92.2)	<0.001
Yes	359 (9.0)	198 (11.3)	108 (6.9)	53 (7.8)	
History of chronic NCDs ^§^					
No	3405 (85.0)	1467 (83.5)	1364 (86.9)	574 (84.7)	0.025
Yes	599 (15.0)	289 (16.5)	206 (13.1)	104 (15.3)	
Income loss during the COVID-19 pandemic					
No	2340 (58.4)	997 (56.8)	964 (61.4)	379 (55.9)	<0.001
Yes	1664 (41.6)	759 (43.2)	606 (38.6)	299 (44.1)	
Financial problems during the COVID-19 pandemic					
No	1992 (49.8)	749 (42.6)	872 (55.5)	371 (54.7)	<0.001
Yes	2012 (50.2)	1007 (57.4)	698 (44.5)	307 (45.3)	
Information exposure during the COVID-19 pandemic					
<1 h/day	1481 (37.0)	683 (38.9)	559 (35.6)	239 (35.2)	0.127
1–2 h/day	1644 (41.1)	701 (39.9)	670 (42.7)	273 (40.3)	
≥3 h/day	879 (21.9)	372 (21.2)	341 (21.7)	166 (24.5)	
Confirmed cases in the community					
No	2562 (64.0)	1006 (57.3)	1069 (68.1)	487 (71.8)	<0.001
Yes	641 (16.0)	321 (18.3)	228 (14.5)	92 (13.6)	
Not known	801 (20.0)	429 (24.4)	273 (17.4)	99 (14.6)	
Quarantine status					
Never	1781 (44.5)	692 (39.4)	754 (48.0)	335 (49.4)	<0.001
Past	1575 (39.3)	786 (44.8)	564 (35.9)	225 (33.2)	
Current	648 (16.2)	278 (15.8)	252 (16.1)	118 (17.4)	
Working from home					
No	865 (21.6)	439 (25.0)	302 (19.2)	124 (18.3)	<0.001
Yes	3139 (78.4)	1317 (75.0)	1268 (80.8)	554 (81.7)	
Fear of COVID-19—NRS (0–10 points), mean (SD); median (range)	6.3 (2.1); 6 (0–10)	6.5 (2.1); 7 (0–10)	6.1 (2.1); 6 (0–10)	5.9 (2.3); 6 (0–10)	<0.001
None/minimal	383 (9.6)	132 (7.5)	147 (9.4)	104 (15.3)	<0.001
Moderate	1681 (42.0)	677 (38.6)	725 (46.2)	279 (41.2)	
Severe	1940 (48.4)	947 (53.9)	698 (44.5)	295 (43.5)	
Perceived risk of COVID-19 infection— NRS (0–10 points), mean (SD); median (range)	5.5 (2.2); 5 (2–10)	5.4 (2.0); 5 (2–10)	5.5 (2.2); 5 (2–10)	5.6 (2.4); 6 (2–10)	0.092
Low perceived risk	767 (19.1)	316 (18.0)	308 (19.6)	143 (21.1)	0.002
Medium perceived risk	1997 (49.9)	927 (52.8)	773 (49.2)	297 (43.8)	
High perceived risk	1240 (31.0)	513 (29.2)	489 (31.2)	238 (35.1)	
Stigma toward COVID-19 infection— COVID-PSS-10, mean (SD); median (range)	24.2 (7.6); 24 (10–50)	24.1 (7.4); 23 (10–50)	24.1 (7.5); 24 (10–50)	24.8 (8.3); 24 (10–50)	0.120
None/minimal	983 (24.5)	425 (24.2)	393 (25.0)	165 (24.3)	0.967
Moderate	1364 (34.1)	605 (34.5)	525 (33.5)	234 (34.5)	
High	1657 (41.4)	726 (41.3)	652 (41.5)	279 (41.2)	
Perceived social support—MSPSS-12, mean (SD); median (range)	59.1 (13.7); 60 (12–84)	54.2 (13.7); 55 (12–84)	61.7 (11.8); 63 (19–84)	65.6 (13.6); 68 (13–84)	<0.001
Low perceived support	226 (5.6)	158 (9.0)	46 (2.9)	22 (3.2)	<0.001
Moderate perceived support	1833 (45.8)	1012 (57.6)	635 (40.5)	186 (27.4)	
High perceived support	1945 (48.6)	586 (33.4)	889 (56.6)	470 (69.3)	

^†^ Data are expressed as the number (percentage) of participants, unless otherwise indicated. ^‡^ The currency exchange in the survey period was 1 USD = 32.5 THB. ^§^ To include diabetes mellitus, hypertension, dyslipidemia, stroke and heart disease, chronic kidney disease, chronic lung disease, and cancer. Abbreviations: BRCS, Brief Resilient Coping Scale—4 items; COVID-19, coronavirus disease 2019; COVID-PSS-10, COVID-19 Public Stigma Scale—10 items; MSPSS-12, Multidimensional Scale of Perceived Social Support—12 items; NCDs, non-communicable diseases; NRS, numerical rating scale; SD, standard deviation.

**Table 3:** Prevalence of mental health issues according to resilient coping status among general population during COVID-19 pandemic in Thailand.

Mental Health Issues	Resilient Coping Status						*p*-Value for Difference
	Low Resilient Coper: BRCS ≤ 13 Points		Medium Resilient Coper: BRCS 14–16 Points		High Resilient Coper: BRCS ≥ 17 Points		
	No. of Cases/ No. of Total	Prevalence Estimated % (95% CI) *	No. of Cases/ No. of Total	Prevalence Estimated % (95% CI) *	No. of Cases/ No. of Total	Prevalence Estimated % (95% CI) *	
Depression (PHQ-9 value of ≥9 points)	952/1756	56.2% (53.0–59.4)	514/1570	36.0% (32.6–39.6)	188/678	29.5% (24.8–34.7)	<0.001
Anxiety (GAD-7 value of ≥8 points)	543/1756	35.2% (32.1–38.4)	277/1570	20.2% (17.3–23.3)	83/678	15.1% (11.5–19.5)	<0.001
Perceived stress (PSS-10 value of ≥14 points)	1553/1756	90.1% (89.1–92.5)	984/1570	64.8% (61.4–68.0)	379/678	56.7% (51.2–62.0)	<0.001
Low well-being (WHO-5 well-being index of <50 points)	1072/1756	64.0% (60.9–67.1)	527/1570	37.2% (33.8–40.8)	151/678	26.1% (21.5–31.3)	<0.001

* Notes: Prevalence is presented as weighted according to the national population and the rate of internet use in Thailand. Abbreviations: BRCS, Brief Resilient Coping Scale—4 items; CIs, confidence intervals; COVID-19, coronavirus disease 2019; GAD-7, Generalized Anxiety Disorder—7 items; PHQ-9, Patient Health Questionnaire—9 items; PSS-10, Perceived Stress Scale—10 items; WHO-5, World Health Organization Five Well-Being Index—5 items.

**Table 4:** Resilient coping status and the risk of mental health issues during the COVID-19 pandemic in Thailand: Multivariable logistic regression model.

Mental Health Issues	Resilient Coping Status *			
	Medium Resilient Coper: BRCS 14–16 Points		Low Resilient Coper: BRCS ≤ 13 Points	
	OR (95% CI)	*p*-Value	OR (95% CI)	*p*-Value
Depression (PHQ-9 value of ≥9 points)				
Model 1 ^†^	1.26 (0.95–1.68)	0.113	2.81 (2.14–3.70)	<0.001
Model 2 ^‡^	1.35 (1.01–1.82)	0.049	2.70 (2.02–3.59)	<0.001
Model 3 ^§^	1.23 (0.91–1.68)	0.182	1.89 (1.39–2.56)	<0.001
Anxiety (GAD-7 value of ≥8 points)				
Model 1 ^†^	1.34 (0.92–1.94)	0.117	2.80 (1.98–3.94)	<0.001
Model 2 ^‡^	1.42 (0.96–2.08)	0.076	2.67 (1.85–3.85)	<0.001
Model 3 ^§^	1.39 (0.94–2.07)	0.101	2.13 (1.45–3.14)	<0.001
Perceived stress (PSS-10 value of ≥14 points)				
Model 1 ^†^	1.34 (1.03–1.76)	0.031	7.04 (5.17–9.59)	<0.001
Model 2 ^‡^	1.40 (1.06–1.84)	0.017	6.54 (4.76–8.97)	<0.001
Model 3 ^§^	1.24 (0.93–1.66)	0.139	4.61 (3.30–6.45)	<0.001
Low well-being (WHO-5 well-being index of <50 points)				
Model 1 ^†^	1.62 (1.21–2.18)	0.001	4.79 (3.60–6.36)	<0.001
Model 2 ^‡^	1.71 (1.28–2.30)	<0.001	4.55 (3.41–6.07)	<0.001
Model 3 ^§^	1.60 (1.16–2.20)	0.004	3.18 (2.31–4.38)	<0.001

* Notes: Using high resilient coper as a reference group, the ORs corresponding to 95% CIs are presented weighted according to the national population and the rate of internet use in Thailand. ^†^ Model 1 adjusted for age and sexual identity. ^‡^ Model 2 adjusted for model 1 plus marital status, education level, religion, occupation, region of residence, living status, personal income, reimbursement scheme, history of mental illness, history of chronic non-communicable diseases, income loss, and financial problems. ^§^ Model 3 adjusted for model 2 plus information exposure, confirmed cases in the community, quarantine status, working from home, fear of COVID-19, perceived risk of infection, stigma toward infection, and perceived social support. Abbreviations: BRCS, Brief Resilient Coping Scale—4 items; CIs, confidence intervals; COVID-19, coronavirus disease 2019; GAD-7, Generalized Anxiety Disorder—7 items; ORs, odds ratios; PHQ-9, Patient Health Questionnaire—9 items; PSS-10, Perceived Stress Scale—10 items; WHO-5, World Health Organization Five Well-Being Index—5 items.

**Table 5:** Ancillary analysis: Multivariable linear regression model results of resilient coping status and the risk of mental health issues.

Mental Health Issues	Resilient Coping Status *			
	Medium Resilient Coper: BRCS 14–16 Points		Low Resilient Coper: BRCS ≤ 13 Points	
	β Coefficient (95% CI)	*p*-Value	β Coefficient (95% CI)	*p*-Value
Depression (lower = better)				
Model 1 ^†^	0.55 (−0.16 to 1.26)	0.132	2.96 (2.25 to 3.66)	<0.001
Model 2 ^‡^	0.74 (0.06–1.43)	0.034	2.71 (2.02 to 3.40)	<0.001
Model 3 ^§^	0.51 (−0.14 to 1.17)	0.126	1.71 (1.01 to 2.40)	<0.001
Anxiety (lower = better)				
Model 1 ^†^	0.47 (−0.11 to 1.05)	0.111	2.35 (1.76 to 2.93)	<0.001
Model 2 ^‡^	0.58 (0.01 to 1.15)	0.045	2.11 (1.52 to 2.70)	<0.001
Model 3 ^§^	0.45 (−0.09 to 1.00)	0.103	1.41 (0.82 to 2.00)	<0.001
Perceived stress (lower = better)				
Model 1 ^†^	1.17 (0.40 to 1.93)	0.003	4.93 (4.19 to 5.67)	<0.001
Model 2 ^‡^	1.31 (0.58 to 2.05)	<0.001	4.53 (3.82 to 5.25)	<0.001
Model 3 ^§^	1.05 (0.34 to 1.77)	0.004	3.61 (2.88 to 4.34)	<0.001
Low well-being (higher = better)				
Model 1 ^†^	−7.68 (−10.18 to −5.17)	<0.001	−20.28 (−22.79 to −17.77)	<0.001
Model 2 ^‡^	−7.86 (−10.26 to −5.46)	<0.001	−18.80 (−21.26 to −16.34)	<0.001
Model 3 ^§^	−6.51 (−8.83 to −4.19)	<0.001	−13.83 (−16.34 to −11.32)	<0.001

* Notes: Using high resilient coper as a reference group, the ORs corresponding to 95% CIs are presented weighted according to the national population and the rate of internet use in Thailand. ^†^ Model 1 adjusted for age and sexual identity. ^‡^ Model 2 adjusted for model 1 plus marital status, education level, religion, occupation, region of residence, living status, personal income, reimbursement scheme, history of mental illness, history of chronic non-communicable diseases, income loss, and financial problems. ^§^ Model 3 adjusted for model 2 plus information exposure, confirmed cases in the community, quarantine status, working from home, fear of COVID-19, perceived risk of infection, stigma toward infection, and perceived social support. Abbreviations: BRCS, Brief Resilient Coping Scale—4 items; CIs, confidence intervals; COVID-19, coronavirus disease 2019; GAD-7, Generalized Anxiety Disorder—7 items; ORs, odds ratios; PHQ-9, Patient Health Questionnaire—9 items; PSS-10, Perceived Stress Scale—10 items; WHO-5, World Health Organization Five Well-Being Index—5 items.

## Data Availability

Data will be shared upon reasonable request and with permission according to the Health Outcomes and Mental Health Care Evaluation Survey Research Group (HOME-Survey) data release policy.

## Supplementary Materials

The following supporting information can be downloaded at: https://www.mdpi.com/article/10.3390/ijerph192013023/s1, Figure S1: Flow Diagram for Study Participation.
