# Supplementary material for: Psychological Resilience and Adverse Mental Health Issues in the Thai Population during the Coronavirus Disease 2019 Pandemic
Source: Int J Environ Res Public Health. 2022 Oct 11;19(20):13023. doi: 10.3390/ijerph192013023 (PMC9602542; doi:10.3390/ijerph192013023)
Supplement: Supplementary file 1 [file ijerph-19-13023-s001.zip › ijerph-1898467-supplementary.pdf]

Online Supplementary Materials

Psychological Resilience and Adverse Mental Health Issues in the Thai Population During the Coronavirus Disease 2019 Pandemic

Chidchanok Ruengorn, Ratanaporn Awiphan, Chabaphai Phosuya, Yongyuth Ruanta, Nahathai Wongpakaran, Tinakon Wongpakaran, Kednapa Thavorn, Surapon Nochaiwong; for the Health Outcomes and Mental Health Care Evaluation Survey Research Group (HOME-Survey)

[*]**Correspondence and requests for materials:**
Kednapa Thavorn, PhD, School of Epidemiology and Public Health, Faculty of Medicine, University of Ottawa, Ottawa, K1G 5Z3 Ontario, Canada, Phone: (613)-7378899 ext 72330, Email: kthavorn@ohri.ca

or

Surapon Nochaiwong, PharmD, Department of Pharmaceutical Care, Faculty of Pharmacy, Chiang Mai University, Chiang Mai 50200, Thailand, Phone: 66899973365, Fax: 6653222741, Email: surapon.nochaiwong@cmu.ac.th

Supplementary Online Content

Figure S1 Flow Diagram for Study Participations

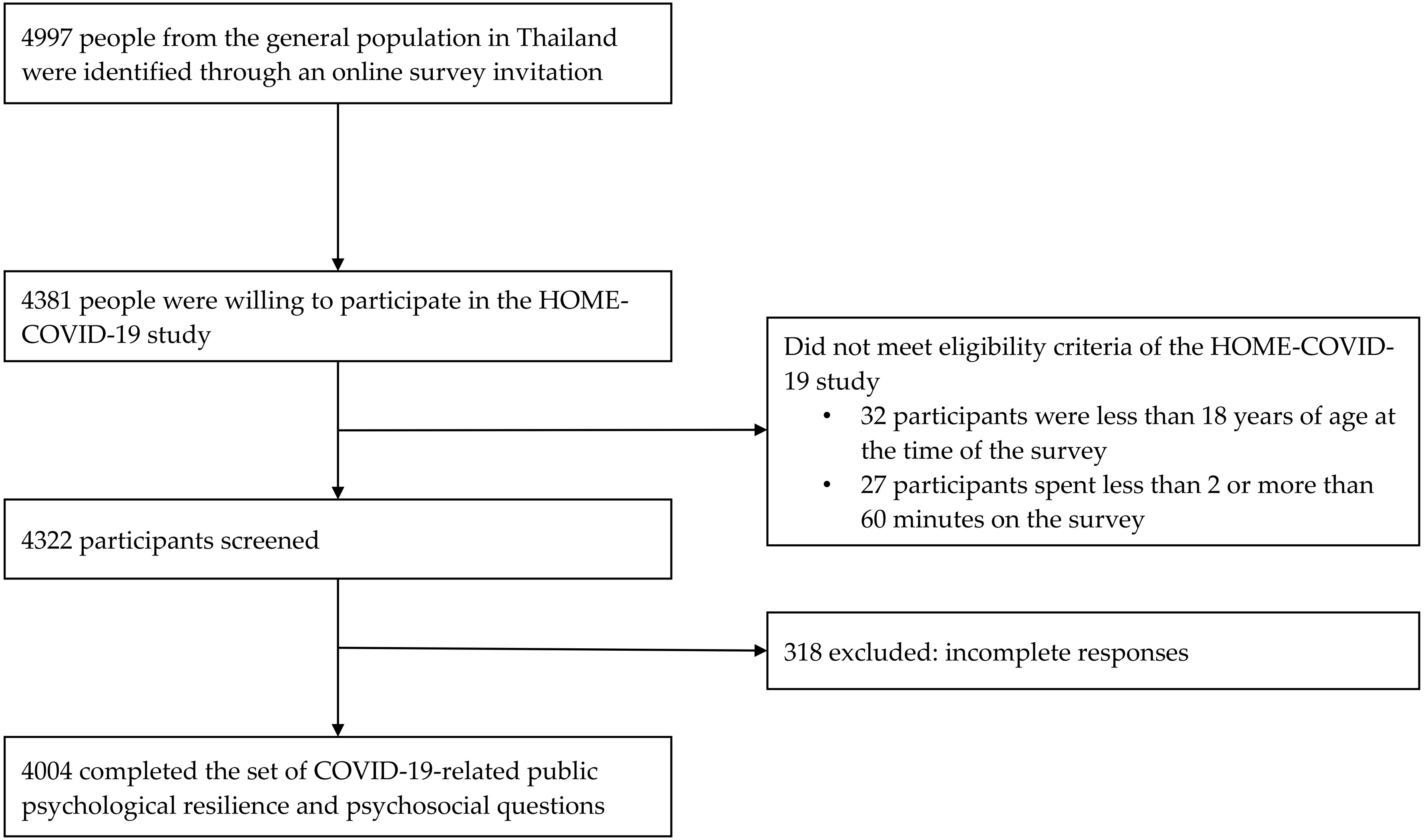

Abbreviation: HOME-COVID-19, The Health Outcomes and Mental Health Care Evaluation Survey Research Group—Coronavirus Disease 2019.
